# Supplementary material for: Patterns of Intron Gain and Loss in Fungi
Source: PLoS Biol. 2004 Nov 30;2(12):e422. doi: 10.1371/journal.pbio.0020422 (PMC532390; doi:10.1371/journal.pbio.0020422)
Supplement: Table S1 — Also available at http://genes.mit.edu/NielsenEtAl/. (4.3 MB ZIP). [file pbio.0020422.st001.zip › NielsenEtAl/html/338.html]

AN3874.1.NCU02550.1.MG03601.1.FG00862.1


```
 CLUSTAL W (1.82) Multiple Sequence Alignments - Introns Inserted


Sequence 1: NCU02550.1	643 aa
Sequence 2: MG03601.1	901 aa
Sequence 3: FG00862.1	893 aa
Sequence 4: AN3874.1	863 aa
Alignment Length: 939 aa
Number Identitical Residues: 199 aa
Alignment Score (without introns) 13376


MG03601.1 	MGPPLDDVVAGMPSALIHAQKIPPANLVADETRFEITSYPSLTQVTKTPATPGQ~VDFVV
NCU02550.1	------------------------------------------------------~-----
FG00862.1 	-MPSDNYNFGDVFQKIYVAKQKPPPIPVHENMKVVLQSSPPLGQVTSVRGR--N~VALTT
AN3874.1  	-------------------------------MPVDLTCYPPLGKVTCLEAD--K0IRFTI
          	                                     . ..  . :   .   .      

MG03601.1 	VLQVHQSRAHDKWQVAVSTSALHSSVWTDSVLASSSAENVPIYLQASDDKSVRKLHFTAS
NCU02550.1	------------------------------MVARQAGSKLP-------------------
FG00862.1 	VLEIPKFRANESWEVSAWHS-VDDAAWKDVPLSSVQEASAPQTLQAFDDSMSR-FYFSTS
AN3874.1  	LIEP---ASQGQYEFQIWHN-IGLSEWAALPLSEYPSAALPALSSRQDPGNRGVFAGEIS
          	  .     :  . .     .    :      ::       *   .  .           :

MG03601.1 	I-SVE~SAVRFTLKFRESPDDPWRFVRDELQLEDGIIVPDH-------PDSLPDDIHDVI
NCU02550.1	--SPQ0SMLNFTIKFRQGPDHDWRWVRNEQGLGDGYIVVDVG----APEGSDDEDLPDLI
FG00862.1 	V-PFE~ASLKFTLKFRHSPDADWRWIRDEQGLNDGLIVNTP-------VGLLSNNLSDLI
AN3874.1  	CPQAR~YAGRFTVRYREGSEGEWRWANKERGCHDGELVIVSRGLSSELALSSAQDFANYF
          	 .  .    .**:::*...:  **: ..*    ** :*    . ::       ::: : :

MG03601.1 	EGLSRHVTIRQAVSQSPATCLWQLDAPIAGVGSSDESALSEVGLGLPWGRHLLR2WFALA
NCU02550.1	HGLNPSLKWSSHLSQCPGTRLWTIEVPVDGAKDDDNSTFSHVPLGIPWG-----~-----
FG00862.1 	PNINSDWTIKSHISQSPGTSLWSLEAVIASANGDD-STYRDITIGTPFG-TFAR2WFSLV
AN3874.1  	DGLSPDIQVEVRKSEAPGAALWNISGPVEEARDGH-SGLLRLPLGTPSS--MSR~FFALA
          	 .:.         *:.*.: ** :.  :  . ... *    : :* * .        :  

MG03601.1 	RTWAPWIAPCHGKAKFDLDRDAVMCSFLN~DQGKHCVLLAVTGTDHVLTG---LRSNDAG
NCU02550.1	-------------------------GFLS2-KGKHLVLLGFAGINNAVDGMTLFRNDEFG
FG00862.1 	RLWSPWLAPRHGKSHFSLDKDGVLCCFLS~PQGKSLVFLAVSGIGHVLPV---FRSESDG
AN3874.1  	RVETSWLGPRQGKDKLNFTEDAILLSFLR~TDGVHVVLLGVT-VDDTLTV---LGSGPAG
          	   :.  ..  ..   .   ..    **   .*   *:*..:  ...:     : .   *

MG03601.1 	KVVLR0IRSDNLAETAAKVIVAVGETFDNASAACVYHARGLLQLDEQQKSSPEGPKQIEN
NCU02550.1	RVTIH0IRNDSAKAGSGTVLVAVGDNFESANAAVMYHARGLVSTADR---AGESSTKLEK
FG00862.1 	KIQVH0ARNDGLSDETAVILVSEGDDFDCVMASVMYHARNLVSVMKQ--AKKEWSHELSA
AN3874.1  	EVVIK~SQNDNATPSRFQVLAATAADFEVATSALIYEARRLVRPYEN------------T
          	.: ::  :.*.       ::.: .  *: . :: :*.** *:   ..             

MG03601.1 	LAADVQPEWMENWFDGLGYC1TWNALGQRLSEEKILDALNTLAENEIMITNLIIDDNWQD
NCU02550.1	LSDNVEANWYENWYDGLGYC1TWNALGQRLTAEKVIRAVDALADNNINISNLIIDDNWQD
FG00862.1 	LVDDFKPEWLEHWFDGLGFC1TWNALGQKLTDQKISDALDKLGEHNIKVSSLIIDDNWQS
AN3874.1  	AQGGPRTQWLSEWYDGLAYC~TWNGLGQDLSEEKILSALDDLKTAGIRIRTLIIDDNWQS
          	   . ..:* ..*:***.:* ***.*** *: :*:  *:: *    * : .********.

MG03601.1 	ISRTGDGQFQYGWNGFEAEPDAFPYGLKATVSSIRSKHKHIQHVAVWHALLGYWGGIAPG
NCU02550.1	IDYHGD-QWQQGWNDFEAEPKAFPNGLTGLVSEIRSKHKNIEHVAVWHALLGYWAGIAPD
FG00862.1 	IDYQGPSQFQYGWKDFEAEPKAFPQGLKATVSHIRKNHPHIQHIAVWHALLGYWGGIAPD
AN3874.1  	LDNEGAGSWHRALTQFEANSKAFPNGLAKAVTTIREQHRNIEYIVVWHALFGYWGGISPE
          	:.  * ..:: . . ***:..*** **   *: **.:* :*:::.*****:***.**:* 

MG03601.1 	GPIANSYKTVEVVR-EEAKRRGFPLGGPMTVIAKEDVNRFYDDFYRFLASTGVDGVKTDA
NCU02550.1	GNLAKRYRTIEVVRGEDSSRKNIPLGGKMTVIAKEDIHKFYDDFYRFLSESGVAGVKTDA
FG00862.1 	GKLAETYKTIEVTR-EDADRRNLPLGGKMTVIAQEDVSRFYNDFYKFLSDAGVDAVKTDA
AN3874.1  	GSLAAIYKTREVALNSTTRP-------SMLTIDPSDIQRFYNDFYAFLSRSGISGVKTDA
          	* :*  *:* **. .. :          * .*  .*: :**:*** **: :*: .*****

MG03601.1 	QFVIDMWIGAGARRELSDAYLDAWTIASLRHFSNRAISCMSMTPHIMFHSQLPRKRPAIP
NCU02550.1	QFMVDTWVSPKVRRELIQPYLDNWLLASLRYFSGRAISCMSMSPQIIFHTQLPRGRPTML
FG00862.1 	QFMIDTWVEASPRRDLINAYLEAWTISTLRHFSAKAISCMSQFPQALFHSQMPTNRPTIL
AN3874.1  	QSFLDLLADPEDRRSYANAYQDAWTISSLRHFGPKAISCMSQIPQTIFHSQLPTNKPTIV
          	* .:*    .  **.  :.* : * :::**:*. :******  *: :**:*:*  :*:: 

MG03601.1 	LRNSDDFTPAIPASHPWHVWTNAHNGLLTQYFNILPDWDMFQTSH----DYSGFHAAARC
NCU02550.1	CRNSDDFFPDVPSSHPWHVWANAHNSLLTQHLNILPDWDMFQTTG----AYAGFHAAARC
FG00862.1 	VRNSDDFFPEIPASHPWHVWTNAHNAIFMSYLNVLPDWDMFQTVH----EYSGFHAAARC
AN3874.1  	VRNSNDFFPDIDDSHTWHVFCNAHNALLTRYLNGLPDWDMFQTLPENGLDYASFHAAARC
          	 ***:** * :  **.***: ****.::  ::* *********  ...  *:.*******

MG03601.1 	VSGGPIYITDVPGEHDKALISEMTGVTPRGKTVIFRTSAHGKSIDQYIGYTDDALLKVGT
NCU02550.1	VSGGPIYITDVPGQYDLDLIKQMTGVTPRGRTVIFRPSVLGRSLDQYVNYDDLSLLKISA
FG00862.1 	ISGGPIYITDVPGEHDMDLIGQMTGLTPQGKTVIFRPSSLGKTVDPYIGYDDDLLLKVGG
AN3874.1  	ISGGPIYITDKPGQHDIPLIKQMTASTIQGTTITLRPDIAARTLDMYHDIKEGHILCVGT
          	:********* **::*  ** :**. * :* *: :*..  .:::* * .  :  :* :. 

MG03601.1 	YHG~GANSGTSMLGIFNVALRPLTDIIPLARFPGTRPQRTYVVRSHGSGRVSPPIEPGTS
NCU02550.1	YNG~RAVTGTPIMGIFNVSGRPLTELIPLARFSGVLPS----------------------
FG00862.1 	HHR1ASHTGYPILGVFNVSSRPLTEFVPLSSFPGVTPG-YYVVRAHTTGKVSQPTTLEGS
AN3874.1  	YHG~RAGSGSGIIGVFNVSNRVESVIIPVADFPGIYDDQEETGYIVRAHRTGRIVGELHS
          	::   : :*  ::*:***: *  : ::*:: *.*             :   .       :

MG03601.1 	ASMLAVSLGVRGYDILSAFPLSCFTSRSG--------------VDVQVANLGLLGKMSGA
NCU02550.1	------------------------------------------------------------
FG00862.1 	GSLFTTSLPVRGFEILSAFSLTPLSSVKH--------------GDVFVANLGLLGKMAGV
AN3874.1  	SSAVSVTLNERRWEVLTAYPVKTLTFKMNSKDKENESSMPTADVSVDVAILGLLRKMTGV
          	.:  : :      .  :: . .  :    :......:: .::. .   :  .   . :. 

MG03601.1 	AAVVS~SDIQQAEGSGRILVHTRVKALGVLD1MSI--------ARDMFATIHGSVMPEET
NCU02550.1	---MC2SDLLEN---GRLFISTRLKALGVLE1S---------------------------
FG00862.1 	AAVIM~NDIKQERHNSRVLIVTRIKAFGTLG1IYISALPNLTIEGDFLITIQEKVIPVHT
AN3874.1  	AALVS~SDIYIED-TGRLRVDVGIKALGVLG~IYFSCLQDWDIDKHFMVLVSGKPVPRKT
          	:: :  .*:     ..*: : . :**:*.*     :.  .             .  .  :

MG03601.1 	VFVSSQDECVLAVDLEAAWKSVGLKAGYSNEVE~LKFYIG~LGNIDSESS
NCU02550.1	-----------------AWKEMGLEAGWSNEVE~VKVYLT~LEK------
FG00862.1 	VAISTVDKHVLEIDIGKAWKDMGLESRWSNDVE0ARAVYR0VSE------
AN3874.1  	IWK--EDGRVLAIDVEEAWHGLGLEAGWGNEVW~ISVLM-~---------
          	      .      .   **: :**:: :.*:*
```
